# Automanejo de la diabetes en personas socioeconómicamente vulnerables: estudio de factibilidad*

**DOI:** 10.15649/cuidarte.3102

**Published:** 2023-12-24

**Authors:** José Camilo Segura Cortes, María Elisa Moreno-Fergusson

**Affiliations:** 1 . Universidad de La Sabana, Chía, Colombia. Email: josecamysc@gmail.com Universidad de la Sabana Universidad de La Sabana Chía Colombia josecamysc@gmail.com; 2 . Universidad de La Sabana, Chía, Colombia. E-mail: mariae.moreno@unisabana.edu.co Universidad de la Sabana Universidad de La Sabana Chía Colombia mariae.moreno@unisabana.edu.co

**Keywords:** Diabetes Mellitus Tipo 2, Enfermería, Automanejo, Educación en Salud, Estudios de Factibilidad, Diabetes Mellitus, Type 2, Nursing, Self-Management, Health Education, Feasibility Studies, Diabetes Mellitus Tipo 2, Enfermagem, Autogestao, Educagao em Saúde, Estudos de Viabilidade

## Abstract

**Introducción:**

La diabetes mellitus tipo 2 es una de las principales causas de morbimortalidad en el mundo, su prevalencia es aún mayor en países de medianos y bajos ingresos. El automanejo es una estrategia que ha demostrado controlar las enfermedades crónicas.

**Objetivo:**

Determinar la factibilidad y eficacia preliminar de la intervención educativa AMAS + Vida enfocada en el automanejo de la salud dirigida a personas adultas con diabetes tipo 2 en una Institución de salud de primer nivel en Neiva, Colombia.

**Materiales y Métodos:**

Estudio de factibilidad, pre test - post test con un solo grupo, para establecer la eficacia preliminar de la intervención. Muestra intencional de 36 adultos con diabetes tipo 2. Los instrumentos empleados fueron ficha de caracterización y escala Partners in Health para medir automanejo.

**Resultados:**

31 adultos con diabetes completaron el seguimiento de 3 meses, mayoría eran mujeres, con bajo nivel socioeconómico y educativo, hubo buena factibilidad de la intervención. Los participantes mejoraron significativamente los conocimientos de la enfermedad (p < 0,001); además hábitos alimenticios (p = 0,001), comportamientos de automanejo de la salud (p < 0,001) y disminución del índice de masa corporal (p = 0,01). No hubo cambios significativos en la actividad física (p = 0,125).

**Discusión:**

Las intervenciones basadas en el automanejo estructuradas bajo la teoría de adaptación a las enfermedades crónicas logran cambios en la promoción de la salud en personas con diabetes.

**Conclusiones:**

La intervención tuvo eficacia preliminar en el grupo estudiado con buena factibilidad. Se recomienda continuar desarrollando estudios de tipo experimental.

## Abstract (en)

**Introduction:**

Type 2 diabetes mellitus is a leading cause of morbidity and mortality worldwide, and its prevalence is even higher in middle- and low-income countries. Self-management is a strategy that has been shown to control chronic diseases.

**Objective:**

To determine the feasibility and preliminary efficacy of the AMAS + Vida educational intervention focused on health self-management in adults with type 2 diabetes in a primary health care facility in Neiva, Colombia.

**Materials and Methods:**

Feasibility study using a one-group pretest-posttest design to determine the preliminary efficacy of the intervention. Purposive sample of 36 adults with type 2 diabetes. The instruments used were a characterization form and the Partners in Health scale to measure self-management.

**Results:**

Thirty-one adults with diabetes completed the 3-month follow-up, most of whom were women with low socioeconomic and educational levels. The feasibility of the intervention was good. Participants significantly improved disease knowledge (p < 0.001), eating habits (p = 0.001), health self-management behaviors (p < 0.001), and decreased body mass index (p = 0.01). There were no significant changes in physical activity (p = 0.125).

**Discussion:**

Self-management-based interventions, structured according to the theory of adaptation to chronic illness, achieve health promoting changes in people with diabetes.

**Conclusions:**

The intervention showed preliminary efficacy in the study group with good feasibility. Further experimental studies are recommended.

## Resumo

**Introdugáo:**

O diabetes mellitus tipo 2 é uma das principáis causas de morbidade e mortalidade no mundo; sua prevaléncia é ainda maior em países de baixa e média renda. A autogestao é uma estratégia que comprovadamente controla doengas crónicas.

**Objetivo:**

Determinar a viabilidade e eficácia preliminar da intervengao educativa AMAS + Vida focada na autogestao da saúde dirigida a adultos com diabetes tipo 2 em uma instituigao de saúde de primeiro nível em Neiva, Colómbia.

**Materiais e Métodos:**

Estudo de viabilidade, pré-teste - pós-teste com grupo único, para estabelecer a eficácia preliminar da intervengao. Amostra intencional de 36 adultos com diabetes tipo 2. Os instrumentos utilizados foram uma ficha de caracterizagao e a escala Partners in Health para medir a autogestao.

**Resultados:**

31 adultos com diabetes completaram o acompanhamento de 3 meses, a maioria eram mulheres, com baixo nível socioeconómico e educacional, houve boa viabilidade da intervengao. Os participantes melhoraram significativamente o conhecimento sobre a doenga (p < 0,001); também hábitos alimentares (p = 0,001), comportamentos de autogestao da saúde (p < 0,001) e diminuigao do índice de massa corporal (p = 0,01). Nao houve alteragóes significativas na atividade física (p = 0,125).

**Discussao:**

Intervengóes baseadas na autogestao estruturadas sob a teoria da adaptagao as doengas crónicas alcangam mudangas na promogao da saúde das pessoas com diabetes.

**Conclusoes:**

A intervengao teve eficácia preliminar no grupo estudado com boa viabilidade. Recomenda-se continuar desenvolvendo estudos experimentais.

## Introducción

La diabetes mellitus tipo 2 (DM2) es una enfermedad crónica grave, en el año 2021, 537 millones de personas entre 20 y 79 años tenían diabetes en el mundo y 6,7 millones de ellas murieron por esta causa y sus complicaciones en ese año. Las personas con DM2 representan el 10,5% de la población mundial en ese grupo de edad y su prevalencia es mayor en los países de ingresos bajos por falta de financiación, tecnología para el diagnóstico y atención integral de la persona^1^.

En Colombia, se reportaron 1.576.508 adultos con DM2 en el 2021, para una prevalencia nacional de 3,11 casos por cada 100 habitantes. Neiva, para el año 2019 fue la ciudad con la mayor prevalencia de diabetes encontrando, 14.073 personas (4,3%) en todo el país^2^. Las personas con DM2 deben modificar su estilo de vida (hábitos alimenticios y actividad física) y adherirse al tratamiento farmacológico para controlar la enfermedad. Sin embargo, el bajo nivel educativo y socioeconómico, la polifarmacia, tener edad avanzada, y la falta de apoyo familiar, se presentan como barreras para un manejo eficaz de la enfermedad^3^.

La Organización Mundial de la Salud (OMS) y la Asociación Americana de Diabetes (ADA) han propuesto el automanejo para el control de las enfermedades crónicas; esta estrategia mejora la capacidad del individuo para comprender la naturaleza de su condición de salud, gestionar y organizar el acceso a su cuidado^4^. Los comportamientos de automanejo de la DM2 son fundamentales porque las personas tienen la responsabilidad de tomar decisiones para modificar su estilo de vida. Estos comportamientos incluyen el conocimiento de la enfermedad, el desarrollo de habilidades, el automonitoreo de la salud, la toma de decisiones, el apoyo social y el trabajo en equipo con el personal de salud^5^.

Las intervenciones enfocadas en el automanejo de la DM2 han demostrado ser costo efectivas y se relacionan positivamente con la adherencia terapéutica y con mejores resultados en la salud^6^^,^^7^, porque empoderan a las personas a participar activamente en su cuidado, adherirse al tratamiento, auto vigilar su condición de salud; promueven la automotivación, y solución de problemas, para que participen activamente en la toma de decisiones relacionadas con su tratamiento^4^^,^^8^.

Desde el punto de vista de enfermería, la teoría de adaptación a las enfermedades crónicas de Buckner y Hayden, derivada del Modelo de Adaptación de Roy, considera el automanejo como una estrategia de afrontamiento y un resultado, por el cual la persona asume la responsabilidad para transformar su estilo de vida. El cuidado de enfermería se enfoca en establecer metas, seleccionar estrategias de intervención, adaptando con flexibilidad las medidas de cuidado, para promover su implementación y la adaptación a la enfermedad de la persona con DM2^9^.

El objetivo de este estudio asociado al proyecto “Automanejo de la diabetes mellitus tipo 2” de la Facultad de Enfermería y Rehabilitación de la Universidad de La Sabana^10^, fue determinar la factibilidad de la intervención educativa AMAS + Vida enfocada en el automanejo de la salud, dirigida a las personas adultas con DM2, socialmente vulnerables, atendidas en una institución de salud de primer nivel en Neiva sobre el conocimiento de la diabetes, los hábitos alimenticios, la actividad física, el índice de masa corporal (IMC) y los comportamientos de automanejo de la salud.

## Materiales y Métodos

Estudio cuantitativo cuasi experimental pre test - post test con un solo grupo, para establecer la factibilidad (tasa de reclutamiento, retención, accesibilidad a los recursos), aceptabilidad y resultados preliminares de la intervención AMAS + Vida basada en el automanejo sobre el conocimiento de la diabetes, hábitos alimenticios, actividad física, índice de masa corporal y comportamientos de automanejo de la salud^11^.

La muestra intencional estuvo constituida por 36 adultos con DM2 entre los 18 y 70 años en condición de vulnerabilidad social (bajo nivel educativo y socioeconómico) atendidos en el servicio de consulta externa de una institución de primer nivel en Neiva - Colombia, durante el segundo semestre del 2021. El tamaño de la muestra cumple con un rango de seguridad para análisis de tamaño de muestra, con una potencia del 90% y un alfa unilateral del 5%, sugerido por Lewis y colaboradores^12^, y se excluyeron las personas con limitaciones cognitivas o físicas, en estado de embarazo o con tratamiento de cáncer.

La información se recolectó con la ficha de caracterización de la población adulta con diabetes mellitus tipo 2 adaptada con previa autorización^13^: se incluyeron los datos sociodemográficos, las características de la enfermedad y su tratamiento, la evaluación de hábitos alimenticios, el cuestionario internacional de actividad física (IPAQ) en versión corta el cual tiene una fiabilidad de 0,65 (r = 0,76; IC 95 %: 0,73-0,77)^14^; la versión en español de la escala de medición sobre el conocimiento de la diabetes en personas con un bajo nivel de alfabetización en salud (SKILLD) con fiabilidad (0,70)^15^; El segundo fue el Instrumento (PIH) para medir comportamientos de automanejo que contiene 12 ítems, para evaluar los factores de conocimiento de la enfermedad, afrontamiento de la enfermedad, manejo de síntomas y adherencia a los tratamientos, con fiabilidad según Alfa de Cronbach de 0,8^16^.

Se aplicó la intervención educativa interdisciplinaria AMAS + Vida fundamentada en el currículo sugerido por la Asociación Americana de Diabetes^17^ y adaptada por Moreno-Fergusson y colaboradores^10^, que incluye los siguientes temas: generalidades de la diabetes, alimentación saludable, actividad física, tratamiento farmacológico, afrontamiento saludable, cuidados para controlar la salud y manejo de las emergencias.

En la adaptación cultural de la intervención participó un equipo interdisciplinario integrado por enfermeros, una fisioterapeuta, una ingeniera de alimentos , una nutricionista y contó con la asesoría de una médica con doctorado en nutrición. Para garantizar la fidelidad de la intervención, se realizó un entrenamiento de un mes con los integrantes del equipo para alinear cada uno de los componentes de esta^18^.

**Figura 1:**
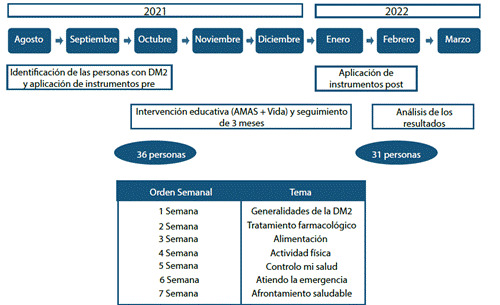
Ruta de la intervención AMAS + Vida

Debido al aislamiento para mitigar el virus del SARS-CoV2, la intervención se entregó de manera individual a través de 7 video llamadas (una semanal) de 30 minutos cada una y la estrategia pedagógica empleada fue la ilustración de experiencias vividas con la diabetes y videos alusivos a los temas mencionados. En la Figura 1 se ilustra la ruta de la intervención AMAS + Vida de este estudio. El responsable de entregar la intervención fue el enfermero líder del proyecto en esta zona del país. 31 participantes se mantuvieron activos en la intervención y durante los 3 meses de seguimiento por vía telefónica, al final del cual se les citó para la segunda medición (ver Figura 1).

Para el análisis de la información, se construyó una base de datos en Excel sometida a verificación y el programa estadístico Statistical Package for Social Science, SPSS® (versión 26) bajo licencia de la Universidad de La Sabana. La información se encuentra almacenada en Mendeley data^19^. Se usó estadística descriptiva (n%, media, desviación estándar, mínimo, máximo) para presentar los datos de las características sociodemográficas, de la enfermedad, del tratamiento, el conocimiento de la enfermedad, hábitos alimenticios, IMC y comportamientos de automanejo; además se realizó prueba de normalidad de Shapiro-Wilk y como no cumplieron con el modelo de normalidad, se aplicó la prueba no paramétrica de Wilcoxon, también se aplicó la prueba de ANOVA para analizar la variable sexo con comportamientos de automanejo. En la variable de actividad física se usó estadística descriptiva (n%) y la prueba estadística de McNemar para muestras relacionadas. Para el contraste de hipótesis se estableció un nivel de significancia de 0,05.

Aspectos éticos: Estudio clasificado de riesgo mínimo^20^ y contó con el aval ético de la Subcomisión de Investigación y Ética de la Facultad de Enfermería y Rehabilitación de la Universidad de La Sabana y el Comité Técnico Científico de la Empresa Social del Estado (ESE) Carmen Emilia Ospina de Neiva. Los sujetos que aceptaron participar firmaron el consentimiento informado.

## Resultados

Los resultados del estudio incluyen la exploración de la factibilidad de la intervención, la descripción de las características sociodemográficas, de su enfermedad y tratamiento, conocimientos de la DM2, hábitos alimenticios, actividad física, medidas antropométricas, comportamientos de automanejo y evaluación de la aceptabilidad.

### Factibilidad de la intervención

Para determinar la factibilidad de la intervención, se tuvo en cuenta los criterios sugeridos por Orsmond y colaboradores^11^.

1. Evaluación de la capacidad de reclutamiento y características de la muestra resultante

Los participantes se reclutaron mediante búsqueda activa en el servicio de consulta externa, cuando asistieron a los controles de riesgo cardiovascular. El reclutamiento duro 8 semanas.

- Disposición de las personas a participar en la intervención

El 92% de las personas tuvo disposición para participar, se determinó por el número de personas que aceptaron participar en el estudio (36) sobre el número de personas elegibles que cumplieran los criterios de inclusión (39).

- Disposición del personal de salud para reclutar participantes

Un profesional de enfermería se encargó de reclutar, aplicar consentimientos informados e instrumentos de valoración.

- Tasa de retención

Se presentó una tasa de retención del 86%, este resultado se estableció del número de personas que finalizaron la intervención (31) sobre el número total de personas que se vincularon al grupo de intervención (36). La retención fue afectada por disponibilidad en tiempo de los participantes por lo que se ajustó la dosis de la intervención por tema y se entregó presencial a 5 personas que no tenían acceso a la virtualidad.

1. Evaluación y perfeccionamiento de los procedimientos y medidas de resultado para la población prevista y el propósito del estudio.

El bajo nivel educativo de los participantes generó dificultades para aplicar la valoración, lo que incrementó el tiempo en recolección de información.

1. Evaluación de recursos y habilidad para manejar e implementar el estudio y la intervención.

La intervención AMAS + Vida se apoyó de herramientas tecnológicas que se adaptaron a las características de las personas, logrando una adecuada motivación y adherencia al programa educativo. La intervención se entregó de manera individual a través de llamadas, video llamadas y presencial en la ESE. Se conformó un equipo de 4 profesionales para dar las sesiones cumpliendo la fidelidad.

1. Evaluación de la eficacia preliminar de las respuestas de los participantes a la intervención.

Los resultados del estudio mostraron cambios estadísticamente significativos con la intervención educativa AMAS + Vida en las variables de comportamiento de automanejo, conocimiento de la enfermedad, hábitos alimenticios y medidas antropométricas (IMC).

### Descripción sociodemográfica, características de la enfermedad y tratamiento de las personas con DM2

Los participantes en su mayoría fueron mujeres (69%) con edad media de 57 años, rango mínimo de 37, y máximo de 70, desviación estándar de 8,23; todos residentes en la ciudad de Neiva; el 94% viven en zona urbana, tienen bajo nivel educativo con secundaria incompleta (69%) y un estrato socioeconómico entre 1 y 2 (95%); La mayoría viven con su familia, en hogares con 1 a 4 personas (89%); el 75% recibe ingresos menores a 1 salario mínimo legal vigente en Colombia, la mayoría son trabajadores independientes (33%) o dedicados al hogar (36%).

Llevaban en promedio 7 años con diagnóstico de DM2, con un rango de menos de 5 años (58%) a 33 años con la enfermedad. La mayoría presentaban comorbilidades, (53%) hipertensión arterial y (41%) dislipidemia. El 64% de los sujetos no tienen glucómetro en casa, el 11% realiza controles diarios de glucometría y el 26% una vez a la semana.

Con relación al tratamiento farmacológico, la mayoría toma hipoglicemiantes orales (94%) de los cuales el 56% tomaba al menos 1 medicamento para la diabetes. El 30% se administraba insulina y en la valoración 3 de ellos tenían lipohipertrofia, debido a errores de aplicación de la insulina. Para el tratamiento de las comorbilidades, el 53% tomaba antihipertensivos, y el 72% tomaba otros medicamentos (ver Tabla 1).

**Tabla 1:** Caracterización sociodemográfica, características de la enfermedad y tratamiento de las personas con DM2

Variable	n(%) 36
Sexo	
Hombre	11 (30,60)
Mujer	25 (69,40)
Ubicación de la vivienda	
Urbano	34 (94,40)
Rural	2 (5,60)
Estrato socioeconómico	
1	15 (41,70)
2	19 (52,80)
3	2 (5,60)
Actividad laboral principal	
Empleado sector publico	2 (5,60)
Empleado sector privado	2 (5,60)
Trabajador independiente	12 (33,30)
Amo de casa	13 (36,10)
Jubilado	1 (2,80)
Desempleado (puede trabajar)	4 (11,10)
Desempleado (no puede trabajar)	2 (5,60)
Nivel educativo	
Primaria incompleta	9 (25,00)
Primaria completa	8 (22,20)
Secundaria incompleta	8 (22,20)
Secundaria completa	6 (16,70)
Técnico	3 (8,30)
Profesional	2 (5,60)
Número de personas que conforman el hogar	
1 a 2 personas	15 (41,70)
3 a 4 personas	17 (47,20)
5 personas o más	4 (11,10)
Ingresos totales del hogar en el último mes	
Menos de 1 salario mínimo	27 (75,00)
1 a 2 salarios mínimos	7 (19,40)
3 a 5 salarios mínimos	2 (5,60)
Tiempo en años de diagnóstico de DM2	
1 a 5 años	21 (58,30)
6 a 10 años	7 (19,40)
11 a 15 años	2 (5,60)
16 o más años	6 (16,70)
¿Con qué frecuencia se toman las glucometrías?	
Nunca	23 (64,00)
Una vez a la semana o más	9 (26,00)
Todos los días	4 (11,00)
¿Se administra insulina?	
Si	11 (30,60)
No	25 (69,40)
¿Toma medicamentos orales para la diabetes?	
Si	34 (94,40)
No	2 (5,60)
¿Cuántos medicamentos orales toma para la diabetes?	
No toma	2 (5,60)
Toma 1 medicamento	20 (55,60)
Toma 2 o más medicamentos	14 (38,90)
¿Toma medicamentos para el control de la tensión arterial?	
Si	17 (47,20)
No	19 (52,80)
Total, de medicamentos diferentes a los hipoglicemiantes	
No toma otro medicamento	10 (27,80)
Toma 1 medicamento	17 (47,20)
Toma 2 o más medicamentos	9 (25,00)

### Conocimiento de la DM2

Los resultados de la comparación de las medidas de tendencia central mostraron una diferencia estadísticamente significativa (p < 0.001) entre el conocimiento que tenían los sujetos sobre la DM2 antes de la intervención Amas + Vida, y el que mostraron 3 meses después.

**Figura 2:**
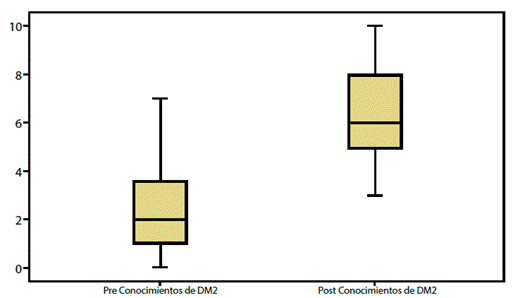
Diagrama de cajas: Conocimientos Pre y Post de la DM2

Al establecer la línea de base previa a la intervención la media fue de 2,32 sobre 10, con una desviación estándar de 1,92; En la medición final después de la intervención se encontró una media de 6,39 con una desviación estándar de 1,82 (ver Figura 2).

### Hábitos alimentícios e índice de masa corporal

**Figura 3:**
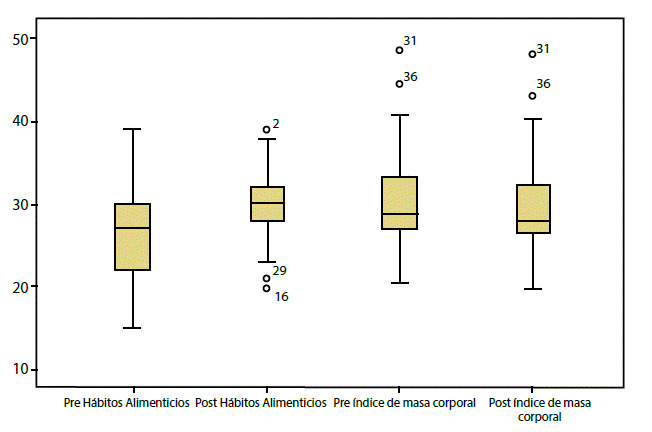
Diagrama de cajas: hábitos alimenticios e índice de masa corporal de las personas con DM2 pre y post

Los resultados de hábitos alimenticios en las medidas de tendencia central muestran una diferencia estadísticamente significativa (p = 0.001) entre las personas con DM2 al inicio del estudio comparados con los tres meses después. En la línea de base la media fue de 25,71, con una desviación estándar de 6,04 y en el seguimiento post intervención se encontró una media de 29,68 con una desviación estándar de 4,40 (ver Figura 3).

Los resultados muestran que el 90,30% de los participantes tenían sobrepeso al iniciar la intervención con reducción del porcentaje al 87,10% después de esta. El seguimiento a los tres meses muestra una diferencia estadísticamente significativa (p = 0,01), entre el IMC pre y post intervención. La media del IMC en la línea de base fue de 30,59 y desviación estándar de 5,98; mientras que la medición post intervención mostró una media de 29,90 con desviación estándar de 5,70 (ver Figura 3).

### Actividad física

Los resultados de este estudio muestran que 4 personas mejoraron el nivel de actividad física después de la intervención. Sin embargo, la prueba relacionada de Mc Nemar muestra que no hay diferencia estadísticamente significativa entre la actividad física de los sujetos del estudio al comparar los resultados del pre test y post test (p = 0,125) (ver Tabla 2).

**Tabla 2:** Tabla de contingencia de los resultados pre y post de la actividad física de las personas con DM2. n(%)

		Post actividad física		Total
		Actividad Física baja	Actividad física moderada o alta	
Pre-actividad física	Actividad física baja	16 (51,60)	4(12,90)	20(64,50)
	Actividad física moderada o alta	0 (0,00)	11(35,50)	11(35,50)
Total		16(51,60)	16(5160)	31(100)

### Comportamientos de automanejo

**Figura 4:**
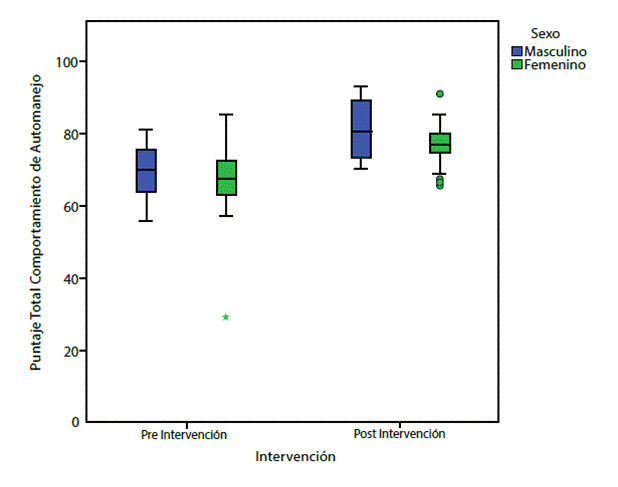
Diagrama de cajas: comportamientos de automanejo de la persona con DM2 comparada con sexo, pre y post

Los resultados de las medidas de tendencia central al comparar el pre test con el post test a los tres meses muestran diferencia estadísticamente significativa (p < 0,001) entre los comportamientos de automanejo de la persona con DM2 pre y post intervención, donde la media de la línea de base fue de 67,97, con desviación estándar 

de 10,04; mientras que en la medición post se encontró una media de 77,93, con desviación estándar de 7,05. Pero al comparar la variable sexo con los comportamientos de automanejo no hay diferencia estadísticamente significativa (p = 1,000) (ver Figura 4).

### Aceptabilidad de la intervención

Respecto a la aceptabilidad de la intervención AMAS + Vida se valoró por medio de frecuencia a través de una pregunta dicotómica por cada parámetro según Sekhon y colaboradores^21^, lo cual mostro que los participantes se sintieron bien durante la intervención (31), no consideraron la intervención como una carga (31), no genero alguna afectación ética (31), comprendieron de manera clara la intervención (31), la intervención no les genero algún costo (27), fue efectiva la intervención (31) y los participantes aplicaron la intervención para la vida diaria (29).

## Discusión

El propósito del estudio fue establecer la factibilidad y eficacia preliminar de la intervención AMAS +Vida de automanejo de la salud para el control de la diabetes mellitus tipo 2 en un grupo de personas atendidas en la ESE de primer nivel en Neiva, utilizando como referente la teoría en enfermería de rango medio de adaptación a las condiciones de salud crónicas de Buckner y Hayden^9^ y los principios de educación de la ADA^17^, sobre conocimiento de la enfermedad y tratamiento, hábitos alimenticios, actividad física, índice de masa corporal y comportamientos de automanejo de la salud.

Respecto a la factibilidad, los resultados mostraron que el 92% de las personas estuvieron dispuestas para el desarrollo del estudio. El reclutamiento duro 8 semanas y hubo una tasa de retención del 86% durante los 3 meses de seguimiento. Con los hallazgos encontrados, se determina que el estudio fue factible y con buena aceptación de los participantes logrando cambios en conocimientos de la enfermedad, hábitos alimenticios, IMC, y comportamientos de automanejo por medio de la educación. Estos resultados guardan similitud con la investigación de García y colaboradores^6^ siendo un estudio piloto que presentó una retención superior al 62% y con comentarios positivos en la aceptabilidad de una intervención basada en el automanejo.

Se destaca que los participantes pertenecían a estratos socioeconómicos y educativos bajos, con ingresos menores a 1 salario mínimo legal vigente en Colombia, con similitud a otros estudios en caracterización socioeconómica del país^3^. Es importante porque la intervención obtuvo resultados positivos y logro una adecuada retención por ser de mínimo costo y adaptada a la población de estudio. Son pocos los estudios que enfatizan dichas características de los participantes, como el de Olry de Labry Lima y colaboradores^22^ el cual hace referencia a una intervención de comunicación de bajo costo para mejorar el automanejo de la diabetes en personas con bajo nivel educativo y socialmente vulnerables en un primer nivel que mejoro las condiciones de salud de los participantes. Aunque la diabetes afecta a millones de personas en el mundo, no diferencia condiciones sociales, es transcendente realizar intervenciones que contextualicen cada población buscando tener mayor impacto en salud en poblaciones vulnerables^23^.

En cuanto a la edad, el promedio fue de 57 años, siendo un grupo todavía productivo con cercanía a la vejez, que realizaban trabajo independiente o labores del hogar, guardando relación con los estudios de Quiñones y colaboradores^24^, Llorente Columbié y colaboradores^25^, por lo que la intervención se desarrolló de manera individual con ajuste a la dosis de cada sesión según la disponibilidad del participante para lograr la adherencia, y cumpliendo con la fidelidad de la intervención. La mayoría de participantes eran mujeres y autores como Mendoza-Catalán y colaboradores^26^ afirman que ellas cumplen mejor con las recomendaciones de alimentación, actividad física y toma de medicamentos en comparación con los hombres por lo que puede existir relación con los resultados presentados. Cabe mencionar que la intervención AMAS + Vida se impartió a ambos sexos en igual condiciones, por lo que se puede proponer futuras intervenciones desde un enfoque de género con el propósito de lograr mayor adaptación.

Se resalta que los participantes de la intervención AMAS + Vida presentaron un cambio significativo entre los comportamientos de automanejo pre y post evaluados por la escala PIH, con aumento en la media de 10 puntos. Por lo tanto, la intervención de manera virtual en la población de bajo nivel educativo y regular acceso a la tecnología fue apropiada para generar cambios en comportamientos y manejo de la enfermedad con logros positivos en el automanejo al igual que en los estudios de House y colaboradores^27^, Von Storch y colaboradores^28^ que se apoyaron de la tecnología. Además, el protocolo educativo de la ADA^17^ fue fundamental para aplicar las sesiones sobre la enfermedad, tratamiento, estrategias psicológicas, y apoyo sobre el estilo de vida con resultados similares a los de Captieux y colaboradores^29^ con mejoras significativas en los comportamientos, tratamiento y estilos de vida de la persona con diabetes.

Por tal razón la intervención AMAS + Vida a través de las sesiones educativas fue eficaz en aumentar el conocimiento sobre la enfermedad y su tratamiento, siendo fundamental para mejorar el proceso de adaptación y con ello los comportamiento de automanejo en salud, al igual como en los estudios Chatterjee y colaboradores^5^, García y colaboradores^30^ donde demostraron que los programas de educación para el automanejo han sido eficaces y facilitan el manejo de la enfermedad, con mejoras en conocimientos, aptitudes y la motivación, lo que se traduce en mejores resultados biomédicos, conductuales y psicosociales. Además, junto con los conocimientos y la escala PIH que evaluaron las habilidades para el tratamiento nuestro estudio mostro mejoría, lo cual según autores de Pamungkas y colaboradores^7^, Amer y colaboradores^31^, se evidencia que la educación en salud sobre la diabetes se asociaba significativamente con un mejor nivel de autoeficacia y cumplimiento de los tratamientos.

Para los hábitos alimenticios los participantes presentaron cambios positivos post intervención con resultados similares a los de Asante^32^, Pamungkas y colaboradores^7^, teniendo en cuenta los factores sociales, cognitivos, de alfabetización y aspectos culturales para generar modificaciones en los ámbitos de salud, siendo estas características importantes al momento de lograr mejoras en el estilo de vida de las personas con DM2. También, en nuestro estudio hubo una reducción en el IMC, sin embargo la población continuo en obesidad en el 87 % de los casos después de la intervención AMAS + Vida, siendo un porcentaje similar al de otras investigaciones^33^^,^^24^^,^^34^, lo que demuestra en general el bajo control metabólico de las personas con DM2 y por tal razón se requiere mayor esfuerzo y seguimiento en futuras intervenciones parar lograr mejores resultados para el control del IMC.

Los resultados de los participantes en el IMC puede tener relación con el bajo nivel de actividad física ya que terminada la intervención AMAS + Vida solo el 48% realizaba actividad física moderada o alta según la escala IPAQ, con igual similitud en el estudio de Vintimilla y colaboradores^35^ siendo determinante mejorar estrategias que aumenten la actividad física de las personas con DM2, y con ello se debe reevaluar nuevas intervenciones que favorezcan los cambios en el estilo de vida.

Al analizar los resultados de este estudio desde la perspectiva de la teoría de adaptación a las condiciones crónicas de salud de Buckner y Hayden^9^, se pudo establecer que los estímulos focales que inciden en el proceso de adaptación son: el diagnóstico de la diabetes y los episodios de alteración de la glicemia que ocurren cuando hay una pérdida del control de la enfermedad; en los estímulos los contextuales, se destacan algunos estímulos comunes como el sexo, la deficiencia en los recursos económicos y la cultura donde influye en la baja actividad física, las pautas de cuidado en la salud y los hábitos alimenticios^36^.

Respecto al proceso de afrontamiento, el conocimiento de la diabetes, las habilidades para aplicar el tratamiento, la interpretación de información sobre la DM2 y la identificación de problemas son fundamentales para el control de la enfermedad. Los resultados de este estudio muestran que en la medida en que los participantes adquirieron un mayor conocimiento sobre estos aspectos, mejoraron los resultados en el automanejo de la diabetes siendo importante la intervención AMAS + Vida en el proceso de adaptación guardando relación con lo que propone Kavuran y colaboradores^37^. Y según las proposiciones de la teoría de Buckner y Hayden^9^, se obtuvo cambios positivos en los comportamientos de automanejo, y con ello el conocimiento de la enfermedad, los hábitos alimenticios, y adquisición de habilidades lo cual se evidenció en la modificación de los patrones de alimentación, IMC y reconocimiento de la información en salud.

Finalmente, se resalta que la intervención AMAS + Vida a través de las sesiones educativas, fue una estrategia relevante en el proceso de adaptación para disminuir los síntomas y complicaciones que puede ocasionar la diabetes, al igual que en los estudios de García y colaboradores^6^ y Chatterjee y colaboradores^5^ donde demostraron que los programas de educación para el automanejo han sido eficaces y facilitan el manejo de la enfermedad, con mejoras en los conocimientos, las aptitudes y la motivación de los personas con diabetes, lo que se traduce en mejores resultados en salud, ya que son intervenciones de minino costo que además permite acoger un equipo multidisciplinario con el fin de lograr un mayor impacto desde el automanejo, adaptando los programas a las características y necesidades socioculturales de la población; dando así una base para elaborar políticas de salud pública desde el primer nivel de atención, enfocadas en el automanejo y prevención de la DM2 y demás enfermedades crónicas que generan altos costos para los sistemas sanitarios.

Respecto a las limitaciones del estudio, se destaca la falta de financiación del proyecto lo cual impidió medir la efectividad preliminar de la intervención en los niveles glicemia y hemoglobina glicosilada siendo datos clínicos objetivos. Así mismo, los sujetos del estudio tuvieron dificultad para conectarse o asistir presencialmente a las sesiones, lo cual ocasionó pérdida de participantes en el seguimiento. Por ser un estudio de factibilidad, los resultados, no son concluyentes. Se requiere la ejecución de estudios experimentales para comprobar la efectividad de la intervención AMAS +Vida, para el control de la DM2.

## Conclusiones

Los resultados de la intervención que se desarrolló con apoyo de la tecnología muestran eficacia preliminar sobre el cambio en el conocimiento de la diabetes y su tratamiento, los hábitos alimenticios, IMC y los comportamientos de automanejo, además tuvo una buena factibilidad y aceptabilidad en población de bajo nivel educativo y socioeconómico, pero se requiere ensayos clínicos aleatorizados a futuro para determinar la efectividad de la intervención.
